# D2R-TED: Data—Domain Reduction Model for Threshold-Based Event Detection in Sensor Networks

**DOI:** 10.3390/s18113806

**Published:** 2018-11-06

**Authors:** Fernando Leon-Garcia, Jose Manuel Palomares, Joaquin Olivares

**Affiliations:** Department of Electronics and Computer Engineering, Edificio Leonardo da Vinci, Campus de Rabanales, Universidad de Córdoba, 14071 Córdoba, Spain; jmpalomares@uco.es (J.M.P.); olivares@uco.es (J.O.)

**Keywords:** WSN, event detection, data compression

## Abstract

The reduction of sensor network traffic has become a scientific challenge. Different compression techniques are applied for this purpose, offering general solutions which try to minimize the loss of information. Here, a new proposal for traffic reduction by redefining the domains of the sensor data is presented. A configurable data reduction model is proposed focused on periodic duty–cycled sensor networks with events triggered by threshold. The loss of information produced by the model is analyzed in this paper in the context of event detection, an unusual approach leading to a set of specific metrics that enable the evaluation of the model in terms of traffic savings, precision, and recall. Different model configurations are tested with two experimental cases, whose input data are extracted from an extensive set of real data. In particular, two new versions of Send–on–Delta (SoD) and Predictive Sampling (PS) have been designed and implemented in the proposed data–domain reduction for threshold–based event detection (D2R-TED) model. The obtained results illustrate the potential usefulness of analyzing different model configurations to obtain a cost–benefit curve, in terms of traffic savings and quality of the response. Experiments show an average reduction of 76% of network packages with an error of less than 1%. In addition, experiments show that the methods designed under the proposed D2R–TED model outperform the original event–triggered SoD and PS methods by 10% and 16% of the traffic savings, respectively. This model is useful to avoid network bottlenecks by applying the optimal configuration in each situation.

## 1. Introduction

Wireless sensor networks (WSN) have been widely used for monitoring environments. Nowadays, WSN are immersed in an exponential growth in many sectors of society such as e-health, industry, agriculture, or smart–cities, among others. Therefore, the amount of deployed devices is huge and increasing each day. However, as more nodes are included, larger data flows are produced. Networks get collapsed as they are not able to handle such amount of information. Thus, network traffic must be reduced.

One of the most common approaches to reduce traffic is by compression. According to Razzaque et al. [1], compression drastically reduces the energy costs, with benefits even greater than linear, since the congestion at the link-level is largely reduced, and, therefore, the effect is wider. In addition, these authors describe the compression in WSN in three levels: Sampling Compression (SC), Data Compression (DC), and Communication Compression (CC). The first type, SC, reduces the amount of sensing (or sampling) while keeping the loss of precision within an acceptable margin. Another strategy, DC, is to transform input data to a new data stream with fewer bits. They are mainly based on the fact that most data streams have redundant values and, thus, more efficient representations can be used. Finally, CC focuses on reducing the amount of communication messages (either transmissions or receptions). This last type is able to provide the largest energy savings, as communications are the most energy-consuming elements in most distributed systems.

Recent works in the state-of-the-art show that there are many lossless compression techniques used in WSN. These techniques are mainly combined with Data Gathering (DG) [2,3,4]. The goal of DG is to efficiently collect all the generated data from multiple deployed sensors in an environment. All of the collected data are usually sent to a central node, commonly called *head cluster*, where data are reconstructed. As identical sensors are placed to capture identical physical variables, there is a possibility of large data redundancy and, therefore, large savings can be achieved if compression techniques are applied. In fact, Data Gathering is combined with Compressed Sensing (CS) [5,6] to produce Compressive Data Gathering (CDG) [7,8,9]. This hybrid scheme interconnects the well-established foundations of CS, based on the representation of a sparse dimensional signal according to fewer projections of the same signal, and DG to collect data efficiently. In order not to lose any data when reconstructing the original signal in the head cluster, the selected CS methods tend to be lossless.

The CS algorithms usually have large computational requirements. However, sensors and their hosting devices are highly constrained in terms of computational power, energy, and bandwidth, as shown in Ref. [10]. Therefore, further lightweight algorithms are included. In this sense, lossy compression methods have usually lower computational requirements. Nevertheless, a trade-off between precision, computing power, and the rest of the constraints must be taken into consideration in the selection of the compression technique.

However, WSNs are not only used to collect data, but to provide responses. These networks are called Wireless Sensor and Actuator Networks (WSAN) [11]. The responses to the actuators are generated automatically according to the system state and the information acquired by the sensors. In these cases, the sensed data can be compressed to reduce the amount of the data sent through the network, but the reconstructed data may not need such accuracy, as long as the obtained response is the same as the one that would be obtained with the original data prior to any compression. Thus, in these cases, lossy compression can be applied, with larger traffic reduction rates and very similar, or even equal, responses.

### Specifying the Scope of Interest

This work considers that the data can be further compressed if it is contextualized, that is, it is known what the extracted data is used for. Therefore, in order to be able to take advantage of the compression methods, this work focuses on deployments where data is acquired and analyzed to provide responses based on the detection of evaluable conditions. Thus, this work is interested in duty cycle-based WSN for condition evaluations on the sensed data. This is quite a common scheme, for instance, WSAN deployments have this type of approach: Nodes that provide information on a given environment, some decisions are made based on all the data acquired and other internal/external variables, and a response is sent to an actuator. This actuator may be applied on the same environment or in other different ones.

In most WSN deployments, the data is sent periodically, in time-triggered schemes. However, there is the possibility of enhancing the transmission process by sending information only when a relevant fact occurs. These mechanisms are included within the event–triggered schemes. Currently, there is much interest in these types of schemes in several fields of interest, for instance, in industrial event–oriented control [12,13], cooperative control of environmental monitoring [14], tracking trajectories of vehicles and robots [15,16], rule-based control of Smart–Homes [17], etc. Currently, large advances have been obtained with diverse variations of the event–triggered schemes, for example, by dynamic adaptive delta mechanisms [18].

The events may provoke responses, which are Boolean in most cases. Therefore, it is possible to analyze the accuracy of the outputs both of the original uncompressed and the event–triggered compressed streams of data. However, the most commonly used metrics in WSN [19] are not suitable by themselves alone for the described task, as they do not take into consideration the accuracy of the outputs in the value domain. In this case, two items must be taken into account: level and transitions. Level is related with the actual Boolean response, while transition deals with the instant where a change in level takes place.

Here, the scenario at hand uses the information to obtain Boolean results. Assuming the applicability of a data compression strategy, the question on its application is how it would affect the result. The analysis of these circumstances from a contextualized perspective, i.e., taking into account the system output, is the main motivation of this work. For example, if a temperature sensor is used to activate a ventilation system when it exceeds a certain threshold, any reduction of the amount of information about the temperature signal can change the triggering pattern of the fans, but it can also reduce the amount of data required for system operation. Therefore, any temperature value that will not change the system state may be ignored because they are not relevant for the output in most cases. This reduction of data is translated into a cost–benefit ratio, which emerges from the contextualized perspective. How to model it is a fundamental motivation of this work.

This paper is structured as follows: Section 2 provides an overview of event detection, compression techniques, and some related work. Section 3 lays the foundation for our proposal. Here, the mathematics behind the data reduction is described. It also describes the metrics for evaluating the data reduction, contextualized to the detection of threshold-based events. Section 4 addresses two case studies to exemplify data reduction with a simple configuration. The reduction process is analyzed in terms of traffic savings and response quality according to the proposed metrics. Section 5 proposes a comparative study to demonstrate the benefits of the model. The properties of the model are used to adapt two known event-based sampling techniques and improve them in the context of the evaluation of Boolean conditions. Finally, some conclusions and future work are pointed out in Section 6.

## 2. Background

WSNs are typically used to collect data from the environment on an ongoing basis. The simplest design is to provide all nodes with a cyclical routine of activation, reading, sending, and deactivation. Over time, the uses of WSNs have multiplied, and the solutions are increasingly sophisticated, generating a large variety of networks according to different criteria [10].

This work focuses on the use of WSNs for detecting threshold-based events, aiming to provide an analytic way to model data compression techniques in that scope, by contextualizing the effects of the compressed data on the Boolean result.

Thus, this work is based on two fundamental concepts: event detection and data compression techniques in WSNs. The following subsections delve into both in order to establish a more comprehensive framework. A third subsection describes the scope of this work as the combination of both concepts, specifying the cases of use and clarifying the specific goal.

### 2.1. Event Detection in WSN

An event is an occurrence or a significant activity that is unusual in relation to normal patterns of behavior [20,21]. Applying the concept to a physical environment, the distributed nature of the sensors combined with their reduced cost and size makes WSNs ideal for monitoring environments in order to detect events.

In Ref. [22], the authors point out the challenges of this task in WSNs by organizing them into four groups: context dependency, application criticality, heterogeneity and overcrowding of data sources, and network topology. In addition, they classify the methods into three groups: statistical, probabilistic, and based on automatic learning techniques and artificial intelligence. According to these authors, the most widely used technique is the detection of events based on a static threshold. It consists of the systematic checking of the condition to know whether the parameter value is within known ranges.

Threshold-based event detection has been used since the earliest declarative systems, which processed Boolean entries known as “facts” [23]. However, the technological framework of the Internet of Things (IoT) has brought about the need to rethink the computation of these Boolean variables in systems where the computational load is distributed in restricted processing cores. On the other hand, it has also brought the possibility of detecting events which are difficult to evaluate with classic centralized systems, e.g., early warning systems for which multiple sensors are required [24] or hazardous environment [25].

Detecting events with WSNs is a problem that has multiple approaches. For example, in Ref. [26], they classify different proposals according to whether the notification decision of the event is made on a sink node or on the node itself. However, the authors of [27] take into consideration the network architecture, the scope of the decision, and the nature of the detection algorithm.

Within the framework of event detection, it is important to note the appearance of Complex Event Processing (CEP) in recent years. This discipline arises from the need to enrich the semantics of conditions in declarative systems, and introduces the concept of a temporal and orderly succession of simple events [28]. The author of [29] proposes various techniques for performing CEP techniques at WSN. As mentioned above, the threshold event detection technique is widely used, a clear example of which is the need for simple event calculation to assess complex events.

The development of concrete applications for the detection of events in WSN or IoT is becoming increasingly importance in recent years [27,30,31,32,33,34,35]. These systems optimize the data transmission related to events by setting filters under different criteria, obtaining large reductions of traffic. As tailor-made solutions, they are an excellent example of good use of the network. However, this type of proposal requires specific analysis of each environment and network deployment in order to establish the general measurement of benefits.

### 2.2. Aggregation and Compression Techniques in WSNs

As mentioned above, a key aspect of this work is to reduce the use of the network accordingly. Here, the most relevant scientific contributions in the field of traffic data reduction WSNs are summarized.

Razzaque et al. [1] provided a survey in which compression techniques were classified into three types: Sampling Compression (SC), Data Compression (DC), and Communication Compression (CC).

Many WSN devices are designed to provide data periodically. Moreover, redundant sensors are usually deployed in an environment. Devices may combine their own data with the data received from others, either providing a stream of modified data, or a value, as the result of a mathematical operation. This is called Data Aggregation (DA). This aggregation results in a reduction of the transmitted data. This strategy can be included within the DC type, as it modifies the data to reduce the amount of data in the network, although some kind of computation and processing is applied in–route on the data.

#### 2.2.1. Communication Compression

An interesting taxonomy of congestion control techniques in WSNs is proposed in Ref. [36]. These techniques are based on the detection or prediction of network congestion conditions and the application of more or less generic strategies to control the situation. This type of proposal is closely linked to infrastructure, and offers routing solutions in most cases. While the purpose of this work is not to control congestion (but to reduce traffic by limiting the loss of response quality), some strategies end up implementing traffic reduction policies to control congestion [37,38,39,40].

#### 2.2.2. Data Compression

Solutions to the congestion problem consider the *network as a service* for data exchange, so they have no control over the sending data. However, there are data reduction techniques that work more closely with information. The authors of Ref. [41] delve deeper into predictive techniques for data reduction in WSNs, classifying models according to the prediction method and the network element where it is applied. The prediction models that are generated in the cluster head nodes [42,43,44], where correlation analysis techniques are used to assess the importance of the data, are particularly noteworthy.

Ikjune et al. [45] proposed an aggregation and compression data method to use in a solar–powered WSN. In it, nodes aggregate data to obtain maximum compression. Data are compressed using a lossless scheme (LZW coding). If the node has more energy coming from its solar panels than it can store in its batteries, aggregated data are sent. Otherwise, it just stops sending data, and just senses its environment, storing the values in its internal memory. This method makes use of the DC type, along with the DA mechanism. However, data are not processed in-route. In addition, the CC is connected to their internal energy status, and not to the Quality of Service (QoS) of the network.

#### 2.2.3. Sampling Compression

As a reference work, the sampling techniques exposed in Ref. [46], a work that is framed in the discipline of control based on events, is worth highlighting. This article discusses the Send–on–Delta [47] technique, which is intended as a fixed increase in the magnitude of a signal whose consecutive samples must be tested in order to be taken into account. In the model proposed in this work, a similar mechanism is proposed but mathematically posed from the domain of system inputs definition, and not subject to static magnitudes.

An important line of research for compressing data is applying PCA to WSN [42,48], in order to reduce the amount of data transferred. There are many sensors providing data that may be correlated. If a PCA is applied to the acquired data space, the dimensionality of the data can be reduced to only a few representative values. The main use for this mechanism is to get rid of several sensors providing similar readings that can be correlated.

Shu et al. [49] proposed DDASA to improve the power efficiency while ensuring the accuracy of the data and have recently stated that “Data reduction exploits the fact that depending on the characteristics of the sampled data within the environment, some data could be redundant”. However, that DDASA algorithm differs substantially from the work presented in this manuscript. In DDASA, the sampling frequency is dynamically and explicitly modified.

#### 2.2.4. Event-Triggered Sampling

In recent years, non-periodic signal sampling techniques have been developed. These techniques propose an alternative approach to determine the sampling instant, instead of the strictly temporal one. For this purpose, strategies based on events intrinsic to the evolution of the data are introduced.

The Send-On-Delta concept (SoD) is the simplest strategy. By means of this scheme, the sampling instant occurs when the magnitude of the difference between the signal value and the previous sample exceeds a confidence interval called delta [47]. This simple concept opened the door to a vast field of research under different topic names, such as event based sampling, Lebesgue sampling, magnitude based sampling, etc. [50,51].

Within the framework of automatic control engineering, event-based sampling techniques are still being developed nowadays. With the mathematical complexity that this discipline requires in terms of stability and convergence of control systems, the sampling criteria have been sophisticated by means of predictive techniques. A simple example of this type of input is Ref. [52], in which a linear predictor is used to establish the sampling instant with the estimated sample, instead of the previous sample.

Considering a distributed scenario where signal samples are sent for processing, these techniques have been considered for traffic reduction, since they limit the amount of network packets tolerating a certain loss of information. An example of this approach is proposed in Ref. [18], where a technique able to select dynamically the limits (delta) according to the available transmission-rate is introduced.

### 2.3. Discussion and Hypothesis

Reducing traffic in sensor networks is a goal that has been addressed from many perspectives. The wide variety of sensor networks results in a broad range of traffic reduction possibilities. To give a few examples: if the network is multihop, the routing policy can contemplate traffic reduction; if the sensor information has some redundancy, traffic can be reduced by aggregating data with space-time criteria; if the network does not have real-time requirements, buffering techniques can be used to collect data on the route, reducing sent packets, etc. In each of these examples, the logic of data reduction is based on a system feature that offers an opportunity to save traffic. This feature is a limiting factor, a requirement. Any data reduction technique is limited by the requirements of its scope.

This work focuses on sensor networks with very specific restrictions. The most important are the topology, the hierarchy of nodes, the timing, and the specific purpose of the data collection. In this framework, there is no information redundancy, there is no multi-hop, and the sensors have very little computational capacity. This means that techniques based on aggregation and compression are not the most suitable for this purpose.

Among the techniques reviewed, event-based sampling ones are applicable to the proposed scenario. They require little computational capacity, and traffic is avoided at the source, thus topology and hierarchy are not a problem. However, event-based sampling approaches are clearly oriented to automatic control engineering, being addressed from the mathematical requirements of this discipline, which requires demonstrating stability, convergence, and other concepts exclusive to its paradigm. This approach is difficult to adapt to other application scenarios, such as many areas of computing where there are no control loops and the versatility of abstraction is fundamental.

A common feature of the revised techniques is that the network has a general purpose, since the context of the data is not considered. In the case of error-tolerant techniques, these are measured as the difference in magnitude between the original data and those obtained after the technique reduction process. This concept of error is intrinsic to the measurement of a real-world signal, and is also decontextualized from the purpose of the information.

Inspired by event-based sampling techniques, this paper proposes a different approach to traffic reduction in sensor networks applied to threshold-based event detection. On the one hand, event-based sampling techniques are reformulated as software filters applied after a regular periodic sampling process, rather than as alternative sampling processes. This means that it is considered a periodic sampling process in the sensors, and the applied techniques will decide sample by sample whether it is sent or not. This approach seems to be simpler and closer to actual sensor network deployments. On the other hand, as part of this reformulation, the necessary abstraction is introduced to adapt the techniques to any data structure, not only to real numbers. This feature makes traffic reduction adjustable to any application, providing versatility. Finally, it is proposed to contextualize the use of information in order to optimize traffic avoidance. As mentioned above, the focus of this work are those networks that evaluate threshold-based events. Based on this knowledge, the concept of error can be redefined according to this context, enabling the definition of traffic avoidance techniques that take into account the system result. As a working hypothesis, let us consider that this approach would provide better cost–benefit rates in terms of traffic savings and errors.

The above approaches are integrated into a general model that can be instantiated for any reduction technique according to its limitations. This model defines the reduction technique using a generic and adaptable mathematical procedure. The effects of this reduction are measured using proposed metrics to take the Boolean result into account. The generic reduction model and the mentioned metrics are detailed in the following section.

## 3. Method

**Figure 1:**
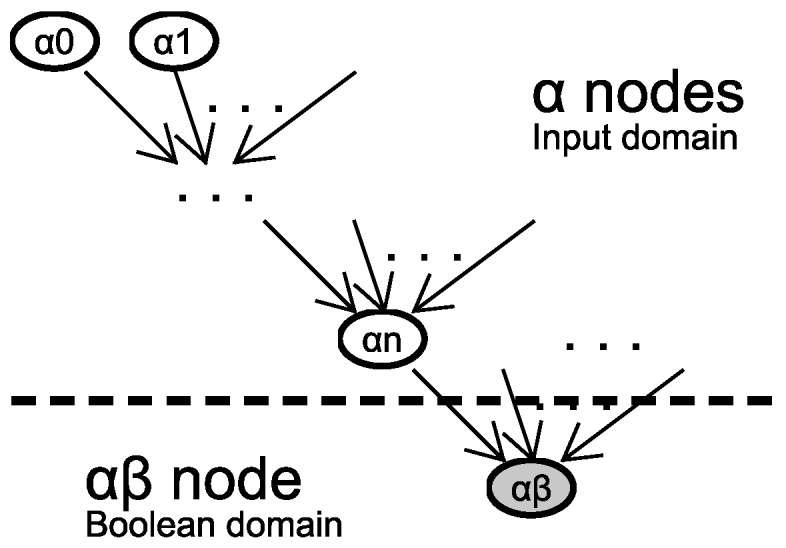
Tree structure.

The study scope is based on those wireless sensor networks which are used for the computation of Boolean conditions. Under the proposed approach, the data flow can be represented as a network structure organized in a tree. The root node performs the calculation of the condition by applying an equation whose inputs are provided by the rest of nodes, directly or indirectly, as shown in Figure 1.

Some simplifications will be considered for this study:

- Condition evaluation is performed periodically with a $t_{s}$ duty cycle period.
- Between two consecutive evaluation instants, the nodes will send an update of their variable only if it has changed.
- All inputs share the same data domain, defined by Φ.

Note that the condition evaluation involves a domain change that takes place at the root node, from $\Phi^{n}$ to B (Boolean domain). This fact contextualizes the use of the input data, and it will allow us to define specific metrics for the analysis of the results.

This section is organized into two subsections. The first one describes a generic mathematical definition for the reduction of input data, with the aim of reducing network traffic. The second one proposes metrics for evaluating the response obtained as a result of this reduction in terms of message savings, precision, and recall.

### 3.1. Data Reduction

One of the motivations of this work is to propose a solution which could be applicable to any system whose purpose and deployment fit in the above-described structure. For this reason, the reduction method is founded on the mathematical description of the domains of the data definition.

Consider the minimum network that fits into the structure described in Figure 1. A single root node called αβ that evaluates a condition whose entries are provided by *n* nodes denoted by $\alpha_{i}$. The entire network can be modeled as a system with *n* inputs and an output in the following terms:

**Definition** **1.** *Let *Φ* be the domain of the values of the system inputs, and let*B*be the Boolean domain {true, false}*.

On that basis, the αβ-node with *n*α-nodes as inputs can be described by a state function $s:\Phi^{n}\to B$ Assuming that the α-nodes obtained their data samples at the same frequency $f_{s}=1/t_{s}$, the system results can be modelled during a time T by Equation (1):

(1) $$\begin{array}{c} s:\Phi^{n}\to B,\forall \varphi \in M_{f\cdot T\times n}\left(\Phi \right),\;\exists b\in M_{f\cdot T\times 1}\left(B\right)\;|\\ b_{x}=s\left(\varphi_{x,0},\varphi_{x,1}\ldots ,\varphi_{x,n-1}\right), \end{array}$$

where $M_{m\times n}\left(D\right)$ is a matrix of *m* rows and *n* columns of elements defined in D, and $a_{x,y}$ is the element at the row *x* and column *y* of a matrix $a\in M_{m\times n}$.

According to the simplification criteria outlined above, α-nodes only send data when information changes. Since the number of unsent packets due to repeated values is unknown, a maximum of $n\cdot f_{s}\cdot T$ network packets are sent in the T analysis period. Based on this approach, a mechanism to promote data repetition is proposed to introduce a reduction in network traffic.

**Definition** **2.** *Focusing on this issue,*R*is introduced as the family of functions that, through a set of parameters defined by *Δ*, translate a set of j–values of *Φ*,*$\Phi^{j}$*, into a set of k–values of *$\Phi^{\prime }$, $\Phi^{\prime k}$, j,k=1…∞. *As*$\Phi^{\prime }$*tends to be a smaller set than *Φ*, these functions are called* reduction functions *in the rest of the paper:*

(2) $$R:\Phi^{j}\times \Delta \to \Phi^{\prime k}\;|\Phi^{\prime }\subseteq \Phi .$$

Thus, giving a function r∈R and the domain of its parameters $\Delta_{r}\subseteq \Delta$, we obtain $\Phi_{r}$, the reduced input domain, as defined below:

$$r:\Phi^{j}\times \Delta_{r}\to \Phi_{r}^{k}\;|\Phi_{r}\subseteq \Phi .$$

**Figure 2:**
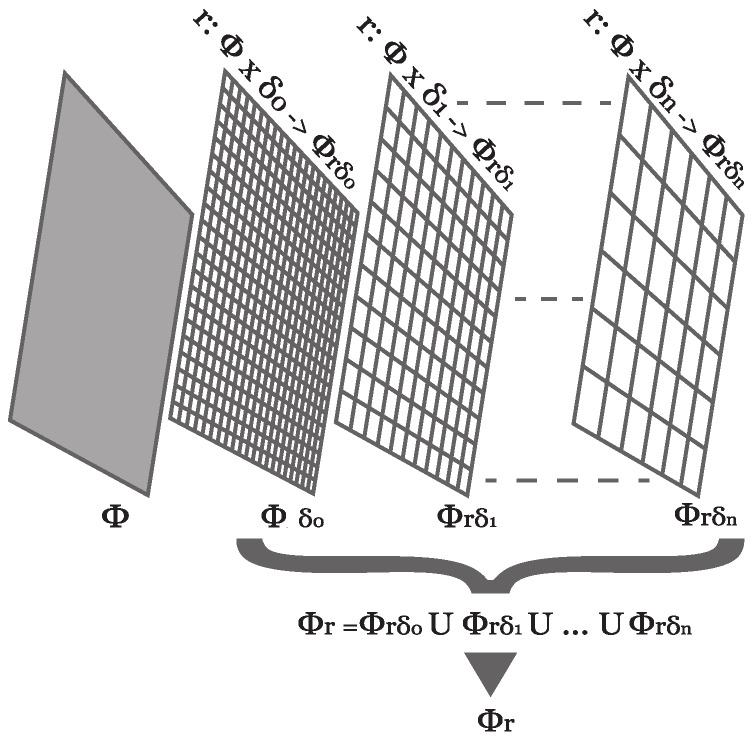
Φ domain reduced to a set of $\Phi_{r\delta }$ domains (*j* = *k* = 1).

Furthermore, every $\delta_{r}\in \Delta_{r}\subseteq \Delta$ generates a different vision of the Φ domain, as described by Equation (3). Figure 2 represents conceptually the reduction of Φ by an r∈R function and its $\Delta_{r}$ parameters:

(3) $$r:\Phi^{j}\times \delta_{r}\to \Phi_{r\delta }^{k}\;|\Phi_{r\delta }\subseteq \Phi_{r}\subseteq \Phi .$$

If an injective *r* function and a set of parameters $\Delta_{r}$ are properly selected, in such a way that the resulting domains have different number of elements, a reduction process is obtained by ordering them by descending cardinality (shown in Figure 2). In this process, different elements of any domain correspond to a single element of the next one.

For the sake of simplicity, but without loss of generality, for the rest of the article, it will be assumed that j=k=1 for the input domains, that is, only one input value is considered in the *reduction functions*. On this basis, given a sequence of values defined in $\Phi_{{r\delta }_{i}}$ with no consecutively coincident values, it is possible that the same signal translated to $\Phi_{{r\delta }_{i+1}}$ may present repetitions, especially if the sequence corresponds to a continuous signal and $\Phi_{{r\delta }_{i}}$ elements are combined in $\Phi_{{r\delta }_{i+1}}$ by proximity. At the same time, reducing the cardinality of the signal domain implies a loss of information that will provoke some alterations in data whose evaluation depends on this signal.

In order to show the general applicability of the proposed model and to help for a better understanding of this concept, the following example has been included. Let us consider the definition of Φ domain as triads of 1-byte-coded integers, representing the RGB color domain. In addition, consider a reduction function (r) that receives as a reduction parameter (δ) the number of bits to encode each integer of the triad.

Thus, for each δ<8, any result of r is within a new domain $\Phi_{\delta }\subset \Phi$, $\Phi_{8}=\Phi$ being the original domain. Defining the set of possible reduction parameters in descending order ($\Delta =\left\{8,7,6,5,4,3,2,1\right\}$) gives a scale of domains by cardinality as described in Figure 2.

**Figure 3:**
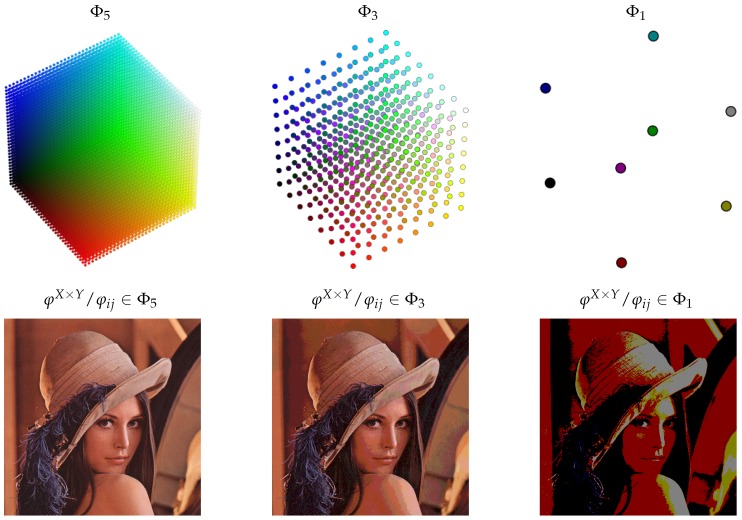
Example of RGB color domain reduction. Components of color coded with 5, 3 and 1 bit.

The top of Figure 3 shows a representation of the reduced domains of the example for δ=5, δ=3, and δ=1, which are $\Phi_{5}$, $\Phi_{3}$, and $\Phi_{1}$, respectively. At the bottom of the figure, different versions of a X×Y matrix of colors are shown. That is an image, and each version is a representation of its pixels (colors) when they are defined in the corresponding domain. In the example described above, each value is a color, and this simplification has allowed the input domain to be represented in a figure. However, let us now consider the domain of X×Y matrix of values defined in Φ as input domain ($\Phi^{\prime }:M_{X\times Y}\left(\Phi \right)$) and the same reduction function and parameters applied to all pixels of the matrix. In this case, a time-ordered sequence of periodic values defined in $\Phi^{\prime }$ is a video, frame by frame. Coming back to the problem of reducing network traffic, it seems reasonable to assume that, if a remote camera implements this reduction procedure without sending duplicate frames, δ parameter will influence the consecutive equivalent frames statistics and, by extension, the network traffic. The question is how big its effect is, and the answer depends largely on what the camera is recording. The variability between consecutive frames will be much lower if the camera monitors a mall at night than if it records a bike ride from the helmet of the cyclist.

The rise of duplicates by reducing the domain cardinality and subsequent result alterations are phenomena with underlying probabilistic logic. However, the analysis would be too complex, since it would have to take into account different aspects, such as signal characteristics, the sequence of domains generated by *r* and $\Delta_{r}$, and all the calculations in which the signal is involved. If the possible reduction of all system signals is taken into account, it is analytically unaffordable. For this reason, this proposal bases the evaluation of the model on empirically obtained metrics detailed in the following subsection.

### 3.2. Metrics

The application of the data reduction method will have two consequences. First, the number of network packets will be reduced due to the promotion of duplicate values in the data streams in the α-nodes. Second, the evaluation of the condition in the αβ-node will suffer alterations due to the loss of information. At this point, analytic methods to quantify both effects will be proposed.

#### 3.2.1. Traffic Reduction Metrics

To obtain the amount of traffic avoided, a ratio between the number of variations present in the sample sequences at the α-nodes applying the reduction with respect to the number of variations that present the same sequences without reduction is proposed. The proposed metric is called *Traffic Saving Ratio* (TSR) and is defined as follows.

**Definition** **3.** *Let*$\varphi \in M_{m\times n}\left(\Phi \right)$*be a matrix of m rows and n columns of values defined in *Φ*,*$\varphi_{x,y}$*being the element of the row x and the column y, and*$m=T\times f_{s}$*. Let us also define*r∈R*as the domain reduction function, and*$\delta \in \Delta_{r}$*the selected reduction parameters. In addition, let be*$\varphi^{\prime }\in M_{m\times n}\left(\Phi_{r\delta }\right)/\varphi_{x,y}^{\prime }=r\left(\varphi_{x,y},\delta \right)$*. The Traffic Saving Ratio (namely,*TSR) *can be defined as the proportion of repetitions of values that appear using the reduced domain. The calculation procedure is stated in Equation* (5)*. Equation* (4) *is required for* TSR *calculation, and it is a key part of the data reduction model, since it implements the equivalence criterion of two values defined in the *Φ* domain. As described in the previous section, this criterion is applied to consecutive values to avoid sending packets in case of equivalence. Note that modifying this equation allows the model to be extended, for example, to fuzzy logic:*

(4) $$\begin{array}{c} \mathrm{eq}\left(\varphi_{1},\varphi_{2}\right)=\left\{\begin{array}{cc} 0, & if\;\varphi_{1}=\varphi_{2},\\ 1, & if\;\varphi_{1}\neq \varphi_{2}, \end{array}\right. \end{array}$$

(5) $$\begin{array}{c} \mathrm{TSR}=1-\frac{\sum_{i=1}^{m-2}\left[1+\sum_{j=0}^{n-1}\mathrm{eq}\left(\varphi_{i-1,j}^{\prime },\varphi_{i,j}^{\prime }\right)\right]}{\sum_{i=1}^{m-2}\left[1+\sum_{j=0}^{n-1}\mathrm{eq}\left(\varphi_{i-1,j},\varphi_{i,j}\right)\right]}. \end{array}$$

#### 3.2.2. Result Evaluation Metrics

To measure the effects on the result, it is interesting to briefly discuss the concept of error in this context. According to the condition Equation (1), the variables are values defined in Φ, and the result is a Boolean value. The error of Boolean values is not quantifiable in terms of magnitude or distance because there are only two possibilities: true or false. In this sense, in each sampling turn, the accuracy of the result can only be measured in terms of correct or incorrect. According to this logic, it is possible to calculate a simple statistic of the number of erroneous results obtained over a period of time. Definition 4 is based on this concept, which can be considered a starting point for the computation of the system precision.

**Definition** **4.** *Based on Definition 3, let us define*$s:\Phi^{n}\to B$*as the condition function implemented in*αβ*. In addition, let*$b,b^{\prime }\in B^{m}/b_{x}=s\left(\varphi_{x,0},\varphi_{x,1},\ldots ,\varphi_{x,n}\right),b_{x}^{\prime }=s\left(\varphi_{x,0}^{\prime },\varphi_{x,1}^{\prime },\ldots ,\varphi_{x,n}^{\prime }\right)$*be the system response using the original and the reduced input domain, respectively. Precision (namely, *P*) can be defined as the proportion of condition results that, when evaluated with input data defined in the reduced domain, match those obtained when using the original domain. Equation* (6) *details the calculation:*

(6) $$\begin{array}{c} P=1-\frac{\sum_{i=0}^{m-1}\left(b_{i}⊕{b^{\prime }}_{i}\right)}{m}. \end{array}$$

**Figure 4:**
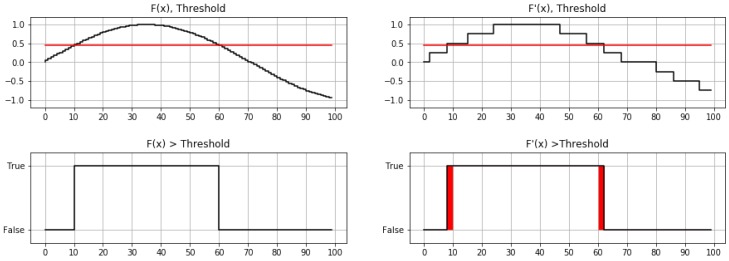
**Upper left**: Original signal F(x), in black; and a constant threshold (Threshold), in red. **Bootom left**: Boolean response of the condition (F(x)>Threshold) applied to the original signal and threshold. **Upper right**: Reduced signal $F^{\prime }\left(x\right)$, in black; and a constant threshold (Threshold), in red. **Bootom left**: Boolean response of the condition ($F^{\prime }\left(x\right)>\mathrm{Threshold}$) applied to the reduced signal and threshold. In red, Boolean variations with the obtained in the bottom left image.

Although this metric is mathematically rigorous, the sample-by-sample evaluated precision is appropriate only if the input data are statistically independent in a sample-by-sample basis. Therefore, each sample is completely independent from the previous one and from the following one. In the scenarios of interest for this research, the input data are samples of continuous signals. Thus, input values correlate with their predecessors, in the same way that the resulting Boolean signal presents transitions between stable levels. In addition, if the reduction method is chosen appropriately, an input value reinterpreted in a reduced domain involves a limited variation from the original one. These facts make it reasonable to consider that most of the errors of the result signal will occur around the transitions, delaying them or bringing them forward in time. To illustrate this, Figure 4 represents on the left side a continuous signal ($F\left(x\right)$) and the result of an inequation applied to it ($F\left(x\right)>\mathrm{Threshold}$). The same signal, but with its samples transferred to a reduced domain, is displayed on the right side. Below, the result of the same inequation for the reduced signal, highlighting the different samples with respect to the result obtained with the original signal is shown. As it can be observed, the differences are grouped around the transitions. This effect has such an important impact that it should be measured.

Based on this idea, the concept of error can be discussed in each application scenario. If the detection of the condition has no real-time requirements, a certain delay in transitions may be tolerable, not being considered an error. For example, returning to the example of the remote camera, if the condition is whether an image recognition algorithm detects a face or not, it may be acceptable for the detection to occur a few frames before or after. To give another example, a network for home automation control that measures the outside temperature to close windows or activate air conditioning can tolerate a delay of some sampling periods to perform the corresponding action.

Here, three metrics that consider this aspect are proposed. These metrics allow for a more descriptive analysis of the consequences of reducing traffic with the proposed method, including a variable, τ, that models the tolerable delay in each scenario. On the one hand, two of the metrics analyze the correspondence between the transitions of the original and the reduced signal. As transitions may appear on one of the signals without matching on the other, this can be analyzed in terms of precision (proportion of obtained transitions that are present in the original signal) and recall (proportion of transitions of the original signal that are obtained). On the other hand, a third metric analyzes the correspondence between signals outside the proximity of transitions, sample by sample.

**Figure 5:**
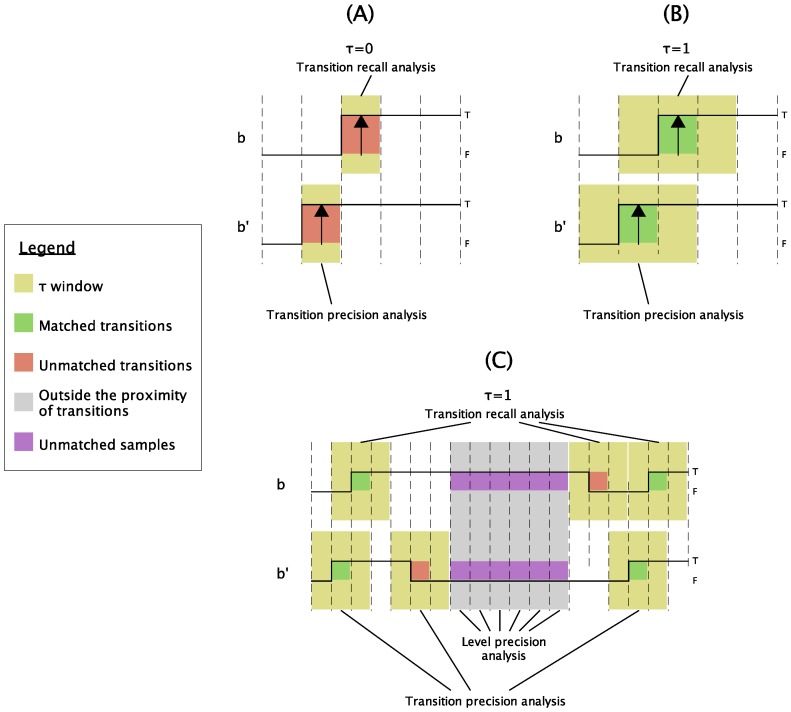
Illustration of the metrics analysis process. (**A**) Transition recall analysis with τ=0. In red, unmatched transitions; (**B**) transition recall analysis with τ=1. In green, matched transitions; (**C**) extended transition recall analysis with τ=1. In green, matched transitions; in red, unmatched transitions; in purple, unmatched samples.

These three metrics are called *Transition Precision*, *Transition Recall*, and *Level Precision*. For the sake of a better understanding of these, Figure 5 represents how the proposed metrics are calculated. For the computation of the transition metrics, the signal value changes are parsed one by one. This process is done with the original signal to obtain the *Transition Recall*, and with the reduced signal to obtain the *Transition Precision*. For each transition, it is checked whether there is an equivalent change in the other signal. By means of the parameter τ, a window of ±τ sampling periods is established, within which the equivalent transition will be considered as valid. In Figure 5A,B, this analysis is represented for τ=0 (the most restrictive case) and for τ=1, respectively. All other signal samples that are not transitions and are not within any ±τ interval are taken into account in *Level Precision* metric. This metric evaluates the occurrence of situations such as those illustrated in Figure 5C, where the reduced signal presents erroneous states over time. To exemplify the importance of this metric, consider that two signals without transitions but with opposite values would present 100% transition precision and recall, but 0% level precision.

With regard to the interpretation of the metrics, if a training signal is properly selected, they can be interpreted as probabilities. Thus, the *Transition precision* is the probability that a transition obtained in the reduced signal would have been obtained without reducing it; the *Transition recall* is the probability that the transitions of the original signal will be detected in the reduced one; and *Level Precision* is the probability that the status of the reduced signal is correct after τ samples since the last transition. The level accuracy has a very interesting interpretation when this is maximum (100%). When this happens, it means that there are no errors outside the ±τ transition intervals, which means that the possible erroneous states of the reduced signal will not cover more than 2τ+1 samples. To understand this, note the upper right side of the illustration (Figure 5C). As it can be seen, with τ=1, a possible transition to an erroneous state that is not detected in the *Level Precision*, could not cover more than three samples, since a fourth erroneous sample would lay outside the ±τ intervals, and would be computed as an erroneous sample.

The three metrics calculations are detailed in Definitions 5 and 6.

**Definition** **5.** *Based on Definition 4, let*$b_{T},b_{F},b_{T}^{\prime },$* and*$b_{F}^{\prime }$*be the resulting signals of applying Equations* (7) *and* (8) *to b and* $b^{\prime }$*, respectively. These signals are transition masks to true and false and are used for the following metrics:*

(7) $$\forall b\in B^{m}\;\exists \;b_{T}\in B^{m}|b_{Ti}=\left\{\begin{array}{cc} b_{i} & if\;i=0,\\ \left(b_{i}⊕b_{i-1}\right)\cdot b_{i} & if\;i>0, \end{array}\right.$$

(8) $$\forall b\in B^{m}\;\exists \;b_{F}\in B^{m}|b_{Fi}=\left\{\begin{array}{cc} |b_{i}-1| & if\;i=0,\\ \left(b_{i}⊕b_{i-1}\right)\cdot b_{i} & if\;i>0. \end{array}\right.$$

**Definition** **6.** *According to Definition 5,*τ∈N*being the time offset tolerance margin in number of samples, let us define the* Transition Precision *(TP) as the proportion of error-free transitions in relation to the total number of reported transitions; the* Transition Recall *(TR) as the proportion of reported transitions in relation to the total number of transitions to be reported; and the* Level Precision *(LP) as the proportion of error-free samples measured outside* ±τ *interval of any transition. The calculation procedures are stated in Equations* (9)–(11):

(9) $$TP=1-\frac{\sum_{i=0}^{m-1}\left({b_{F}^{\prime }}_{i}\cdot \left({b_{F}^{\prime }}_{i}⊕min\left(\sum_{j=i-\tau }^{i+\tau +1}{b_{F}}_{j},1\right)\right)+{b_{T}^{\prime }}_{i}\cdot \left({b_{T}^{\prime }}_{i}⊕min\left(\sum_{j=i-\tau }^{i+\tau +1}{b_{T}}_{j},1\right)\right)\right)}{\sum_{i=0}^{m-1}\left({b_{F}^{\prime }}_{i}+{b_{T}^{\prime }}_{i}\right),}$$

(10) $$TR=1-\frac{\sum_{i=0}^{m-1}\left({b_{F}}_{i}\cdot \left({b_{F}}_{i}⊕min\left(\sum_{j=i-\tau }^{i+\tau +1}{b_{F}^{\prime }}_{j},1\right)\right)+{b_{T}}_{i}\cdot \left({b_{T}}_{i}⊕min\left(\sum_{j=i-\tau }^{i+\tau +1}{b_{T}^{\prime }}_{j},1\right)\right)\right)}{\sum_{i=0}^{m-1}\left({b_{F}}_{i}+{b_{T}}_{i}\right),}$$

(11) $$LP=1-\frac{\sum_{i=0}^{m-1}\left(\left(b_{i}⊕b_{i}^{\prime }\right)\cdot \left|min\left(\sum_{j=i-\tau }^{i+\tau +1}\left({b_{T}}_{j}+{b_{F}}_{j}+{b_{T}^{\prime }}_{j}+{b_{F}^{\prime }}_{j}\right),1\right)-1\right|\right)}{\sum_{i=0}^{m-1}\left(\left|min\left(\sum_{j=i-\tau }^{i+\tau +1}\left({b_{T}}_{j}+{b_{F}}_{j}+{b_{T}^{\prime }}_{j}+{b_{F}^{\prime }}_{j}\right),1\right)-1\right|\right)}.$$

As it can be seen, to characterize a system with these metrics, the non–reduced result is required. This implies the need for training data. The effectiveness of the method will depend on how well these data represent the overall behavior of the system.

Regarding the quality of the training data, it is reasonable to assume that the most favorable scenario is the evaluation of conditions based on periodic pattern parameters. For example, parameters related to biological activities influenced by circadian cycles, or meteorological parameters that depend on natural periods. However, since the circumstances that lead to traffic avoidance are not necessarily permanent, we suggest that the entire time of the unrestricted traffic network may be used to fine-tune the metrics to obtain the most reliable model possible.

## 4. Case Study

This section includes two experimental scenarios in which the model is applied and analyzed. Through these use cases, two objectives are intended. First, to exemplify the application of the model over actual and extensive data. Secondly, to test the potential use of the technique to reduce the traffic of a network in a saturation situation, knowing what proportion of traffic will be avoided and how many errors will be introduced into the system.

For these purposes, the data signals from the public repository of the Italian meteorological network Arpa Piemonte [53] are used. This repository contains nine years of hourly data from several stations, and has been considered adequate due to the abundance of quality data.

Each use case presents an experimental scenario consisting of a conditional expression involving signals from the repository. Each signal represents a network sensor, and each signal sample represents a sent data packet. The details of the reduction according to the model requirements are described for each scenario. In order to analyze the potential on–demand application of the model (the second objetive), the signals from the repository are split into two periods. The first period corresponds to the first year of data and is considered as a training period. It is assumed that during this period the data reduction process is modeled according to the results obtained. The remaining eight years are considered the test period. During this period, it is assumed that the network suffers saturation conditions, and the data reduction is applied according to the results of the training period. The comparison of the proposed metrics obtained in each of these periods constitutes an analysis of the repetitiveness of the model and, therefore, of its potential use on demand to reduce traffic knowing the consequences, which is the working hypothesis of these experiments.

First of all, let us briefly summarize the proposal to contextualize the study cases: the starting point is a network of sensors whose data is collected by a central node that evaluates a Boolean condition (called *s* function in this work). The model defines a procedure for reducing network packets by promoting repeated values, which are not sent. The starting point is an input data domain (Φ), which, using a reduction function (r) and a set of parameters ($\Delta_{r}$), gives rise to a domain scale $\Phi_{r}$ (one for each $\delta_{r}\in \Delta_{r}$ parameter) with different cardinalities. Using a set of ordered values defined in Φ, the reinterpretation of this information in any $\Phi_{r_{\delta }}$ ($\in \Phi_{r}$) domain has two consequences: the promotion of duplicate values and the loss of information. Metrics have been proposed to model these effects.

According to the above, the model introduces some generalities that must be defined for each scenario: (1) input data domain Φ, (2) condition expression *s*, (3) sensor data for training and testing, (4) reduction function *r*, and (5) set of parameters for the reduction function Δ. These aspects are specified for each case study, which are detailed in the following subsections.

### 4.1. Case Study I

For this experimental environment, the model has been adapted for a condition tree involving two temperature sensors ((1) Φ=R ) and the sink node. The sink node periodically collects the information and checks whether one temperature is higher than the other ((2) $s\left(\varphi_{1},\varphi_{2}\right)=\varphi_{1}>\varphi_{2}$). The model metrics are calculated for two segments of these signals. The first segment is the first year of data, and corresponds to the time window considered for training. This training period is used to characterize the cost–benefit ratio in terms of traffic savings and errors introduced by the reduction technique in its different configurations. The second segment includes the remaining eight years, and its results are used to analyse the repeatability of the results obtained in the training period. Results of both periods are compared to discuss the hypothesis. The reduction function truncates the values to a multiple of a parameter, which is the temperature resolution ((4) $r\left(\varphi ,\delta_{r}\right)=\mathrm{Integer}\left(\frac{\varphi }{\delta_{r}}\right)\cdot \delta_{r}$ ). The set of possible resolutions (Δ) is $\left\{0.1,0.25,0.5,1.0,2.5\right\}$, 0.1 being the original resolution (non-reduced case) (5). This scale is typical for oscilloscopes and measuring instruments. Regarding the sensor data, from the repository mentioned above, the temperature data from the Alessandria and Montaldo Scarampi stations were used. The selection criterion corresponds to the proportion of true and false in the results of the evaluation of the previously stated condition. Specifically, both signals are composed of 78,840 samples; the signal resulting from the condition, sample by sample, presents 7625 transitions; and the true and false balance is 53%/47%, respectively. For each $\delta \in \Delta_{r}$, the values of the metrics given in Section 3.2 have been calculated for both training and experimental data.

The results are presented in a table for each metric. By columns, $\delta \in \Delta_{r}$ parameters from δ=0.25 because δ=0.1 models the original system state, with no reduction, in which case TSR = 0, LP = 1, TP = 1, and TR = 1. The last two rows show the absolute and relative error for each δ value (ϵ and $\epsilon_{r}$). In the case of LP, TP and TR, these errors are averaged ($\bar{\epsilon }$ and $\bar{\epsilon_{r}}$ ) and represented with standard deviation (σ). In addition, each τ value results in a row, and the last two columns represent the absolute and relative error averaged by each τ, with standard deviation as well. The cells in the body of the tables show two values separated by a slash (/): on the left, the result of the calculation with the training data, and on the right of the slash, the results applied to the experimental data (not including the data used for training). Relative errors are calculated considering the value obtained with the training data as the actual value.

Table 1 shows the traffic saving ratio metric (TSR), which has been calculated according to Equation (5). Table 2 shows the level precision, according to Equation (11). Table 3 shows the transition precision, according to Equation (9). Finally, Table 4 shows the transition recall, according to Equation (10). The last three metrics have been calculated with an offset tolerance interval (τ) from 0 to 9. The results are discussed below.

In general, the metrics calculated for the training data fit well with the calculations made for the experimental data. Absolute errors do not present a proportionality relationship with the parameters δ and τ, except for LP metric (Table 2), whose error increases as δ increases too. The largest errors in magnitude are presented by transition precision metric (TP, in Table 3), reaching 0.033 in the worst case. In addition, the greatest relative error is experienced by traffic saving ratio metric (TSR, Table 1), reaching 7.46% in the worst case scenario. As the magnitude of the current value (0.201 in the worst case) is close to 0, the relative error is high. Absolute errors in TSR are also reduced. It seems reasonable to conclude that the training data faithfully represent the experimental data, taking into account the magnitude of errors and that the training data consists of one year of data against eight years of experimentation.

As it was expected, the larger the δ, the larger the traffic saving (TSR, Table 1). Transition precision (TP) and recall (TR) metrics can be interpreted as a probability, since they are obtained from the casuistry of the training data. With this perspective, transition precision is the probability that a transition obtained with reduced input data according to δ would have been detected with non-reduced data with a time offset of no more than ±τ samples. In turn, transition recall is the probability that a transition that would have been detected with non-reduced data will be detected with reduced data according to δ with a time offset of no more than ±τ samples. It is interesting to note the significant increase in precision and recall experienced when comparing an absolutely rigorous approach, without tolerating delays (τ=0), to a more flexible approach tolerating delays (τ>0). The most pronounced increase occurred in the change from τ=0 to τ=1, which suggests that a large part of the transitions that are not instantly detected are detected indeed with a temporal delay of one sampling period. This aspect confirms the grouping of errors around transitions, an effect that is illustrated in Figure 4.

Since it is a demonstrator, the numerical data does not deserve further attention. However, the potential of this proposal lies in the interpretation that can be made of its results. At this point, let us exemplify what the model provides to reduce network traffic. Suppose that the network in which this condition is implemented is overloaded for some reason, and it is urgent to reduce its traffic load by 15%. Also assume that the application tolerates a time delay of three sampling periods (τ=3). An operator or algorithm that controls the model will have the following information (involved values are marked in bold in the tables):

- Table 1 reveals that δ=0.5 will reduce traffic by 20.1%.
- According to Table 3, each transition notified will have a 90.6% chance of being correct.
- Likewise, according to Table 4 and under the same conditions, 94.7% of the transitions that would have been detected without reduction will be detected.
- Table 2 reveals that 100% of the samples outside transitions ±τ sampling periods will be correct. This means that there are no erroneous values that remain more than seven samples over time (2τ+1).

These interpretations are based on the values obtained from the training data. Assuming that the reduction is carried out with these parameters during the whole period of experimentation, the following would have been achieved:

- Reduced network traffic by 18.6% (1.5% lower than expected).
- 91.2% of the transitions detected were correct (0.6% higher than expected).
- 96% of the transitions that would have been detected without reduction were detected (1.3% higher than expected).
- As it was expected, there were no erroneous values that remain more than seven samples over time.

### 4.2. Case Study II

This case study is based on a network of four sensors, two temperature sensors, and two wind speed sensors. Each pair of temperature and speed sensors is used to calculate wind chill (according to the classic Steadman formula [54]) and to compare them. It is intended to enrich the previous demonstrator with a more complex application example. The parameters of the model are as follows:

1. Input domain: Φ=R.
2. Condition state function: $s\left(t_{1},v_{1},t_{2},v_{2}\right)=\left(1.41-1.162\cdot v_{1}+0.98\cdot t_{1}+0.0124\cdot {v_{1}}^{2}+0.0185\cdot v_{1}\cdot t_{1}\right))>\left(1.41-1.162\cdot v_{2}+0.98\cdot t_{2}+0.0124\cdot {v_{2}}^{2}+0.0185\cdot v_{2}\cdot t_{2}\right))$. This is the classic Steadman formula.
3. Input data: all data has been extracted from the repository of the Italian weather network Arpa Piemonte [53], used in the previous case study. Data from sensors $t_{1}$ and $v_{1}$ are extracted from the weather station located in Cameri. The data for $t_{2}$ and $v_{2}$ correspond to the station located in Boves. The training data correspond to the first year of data, the experimental data are the rest. These four signals are composed of 78840 samples; the signal resulting from the condition, sample by sample, presents 14015 transitions; and the true and false balance is 58%/42%, respectively.
4. Reduction function: $r\left(\varphi ,\delta_{r}\right)=\mathrm{Integer}\left(\frac{\varphi }{\delta_{r}}\right)\cdot \delta_{r}$.
5. Reduction parameters: $\Delta_{r}$ = $\left\{0.1,0.25,0.5,1.0\right\}$.

**Figure 6:**
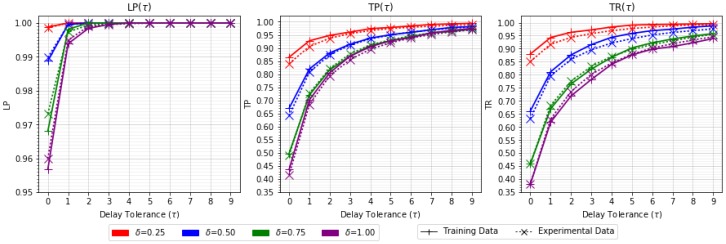
Case study II. LP, TP, and TR metrics for training and experimental data.

From the results obtained, the traffic saving ratio metric (TSR) is represented in Table 5, similar to the previous study. Level precision (LP), transition precision (TP), and transition recall (TR) metrics are shown in graphs, presented in this order from left to right in Figure 6. The graphs show the τ–dependence of the metrics for each δ∈Δ (differentiated by color) with training and experimental data (differentiated by line type).

The results show that the training data fits well with the behavior of the system. The graphics offer a visual perspective that reveals the horizontal asymptotic shape of all curves, in the form $y=1-A\cdot e^{-B\cdot x}$. This fact demonstrates from another perspective the grouping of errors around transitions.

## 5. Comparative Test

In order to adjust the reduction models according to the context in real time, the proposed method enables a scale of reduction configurations. Specifically, this work focuses on the context of Boolean expression evaluation. In this framework, the error according to system output is measured by specific metrics defined in Section 3.2.

This case study addresses a comparative analysis to understand the potential of contextualization. To this end, two known event-driven sampling techniques have been defined according to the model specifications. Then, for each of these techniques, an adaptive version has also been defined for comparison with the original. Adaptive versions use the reduction parameter scale provided by the model to conveniently optimize data reduction based on context. The following subsections detail the aspects of the experimental scenario.

### 5.1. Increasing Linear Threshold ‘K’

**Figure 7:**
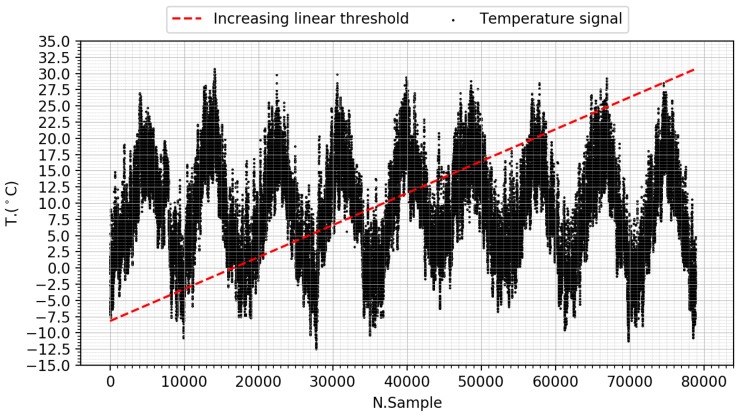
Representation of increasing linear threshold ‘K’, defined for the temperature signal extracted from the Oropa station.

In this case study, temperature signals from the repository of the Italian weather network Arpa Piemonte [53] have been loaded to evaluate the expression $T_{i}\left[n\right]>K_{i}\left[n\right]$ where *i* identifies each station. $K_{i}$ is a linear expression generated for each station according to Algorithm 1, ranging from the minimum to the maximum value of the signal, to eliminate possible biases in experimentation. Figure 7 represents the temperature signal corresponding to the Oropa station and the linear threshold K generated for it.

**Algorithm 1:**$K_{i}$ Generation Method

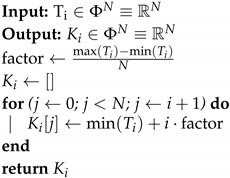



### 5.2. Send–on–Delta and Predictive Sampling

In recent years, non-periodic sampling methods have been developed. These approaches are not based on a regular period of time to report a new sample, but on an event intrinsic to the signal itself. This concept has received different names: event based sampling, Lebesgue sampling, magnitude based sampling, etc.

Send-on-Delta (SoD) [47] is the simplest well known approach. Using the SoD concept, a continuous-time bandlimited signal x(t) is sampled and a new report is sent, when the value of the physical variable being sensed deviates from the value included in the most recent report by an interval of confidence ±Δ. Many other proposals are extended versions of this one, introducing estimation strategies to enrich this event condition detection. Predictive Sampling (PS) [52] was proposed as a simple example of this case, in which a linear predictor is used to trigger sampling if the value of the measured variable deviates from the estimated value by ±Δ.

Most event-based sampling techniques can be easily adapted to the definition of the proposed model if they are understood as filters. That is, it must be assumed that there is an internal process of periodic sampling in the sensors, after which the corresponding technique is applied to decide whether the samples are sent or not. For this scenario, two techniques have been adapted to the model: (a) SoD, and (b) Extended SoD with a linear predictor, which is called Predictive Sampling (PS). The prediction is carried out by linear regression by least squares of the N last samples. This method does not require much computing power, which enables its implementation in small sensors. Algorithms 2 and 3 correspond to the reduction functions for SoD and PS, respectively.

**Algorithm 2:** SoD Reduction Function

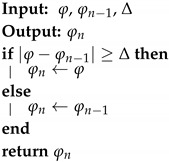



**Algorithm 3:** PS Reduction Function

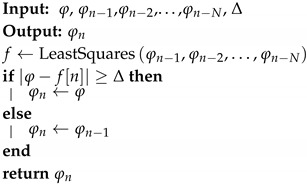



### 5.3. Adaptive Versions of SoD and PS

According to Equations (2) and (3), giving a reduction function r∈R, $\Delta_{r}$ is a set of reduction parameters, where each $\delta_{r}\in \Delta_{r}$ generates $\Phi_{\delta_{r}}$ data domain, with less cardinality than the original input domain, Φ. The definition of the $\Delta_{r}$ set enables the implementation of a reduction scale change heuristic that opportunistically adapts to the context. Within this framework, a scale of reduction configurations and a simple method for its variation at runtime has been proposed in this subsection.

Previously, Algorithms 2 and 3 define the reduction function for the classic SoD and PS methods, respectively. These methods are evaluated in the sensors, which are in charge of filtering and sending the new samples of its signal. The reduction parameter change heuristic is a function located in the sink node, which is the node that uses the data for Boolean calculations and therefore provides context. This implies that each change in the reduction parameters implies a network packet sent from the sink node to the nodes. This traffic will be taken into account in the experiment, by adding the number of sink–to–sensor packets to the numerator of the Equation (5).

The adaptive versions of SoD (A-SoD) and PS (A-PS) implement the same reduction functions defined in Equations (2) and (3), respectively. $\Delta_{r}$ is an ordered list of confidence intervals (different Δ values). The selection of parameter D is made according to Algorithm 4, which applies a distance criterion between the value of the signal and the threshold. In the algorithm, D is an ordered list of distances in magnitude corresponding to each $\delta_{r}\in \Delta_{r}$.

**Algorithm 4:** Reduction Parameter Change Heuristic

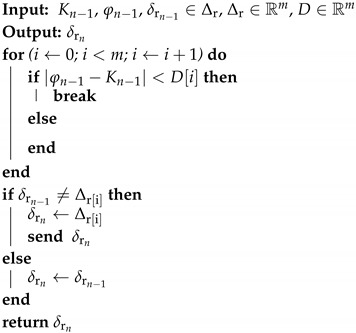



### 5.4. Experiment Configuration and Results

The case study description leaves some parameters unspecified. In each of the techniques described (SoD, PS, A-SoD, and A-PS), the following remains to be determined:

- SoD
  - Δ: Confidence interval in magnitude.
- PS
  - Δ: Confidence interval in magnitude.
  - *N*: Number of previous samples for linear regression by least squares.
- A-SoD
  - $\Delta_{r}$: Ordered list of confidence intervals $\delta_{r}$ in magnitude.
  - *D*: Ordered list of distances between signal and threshold, each distance corresponding to a $\delta_{r}\in \Delta_{r}$ parameter.
- A-PS
  - $\Delta_{r}$: Ordered list of confidence intervals $\delta_{r}$ in magnitude.
  - *D*: Ordered list of distances between signal and threshold, each distance corresponding to a $\delta_{r}\in \Delta_{r}$ parameter.
  - *N*: Number of previous samples for linear regression by least squares.

Parameters Δ, *N*, $\Delta_{r}$, and *D* are established below, and applied indistinctly to the techniques that require them:

- Δ=0.5,
- N=5,
- $\Delta_{r}=0.1,0.5,1.0,2.5$,
- D=2,5,8,11.

This configuration has been determined by trial and error with one year of data prior to the experiment. Parameter optimization requires in-depth analysis beyond the scope of this work. This case study aims to test the adaptability enabled by the proposed model. It is important to remark that the methods compared here are only examples of the use of the model, which is the main contribution of this work.

**Figure 8:**
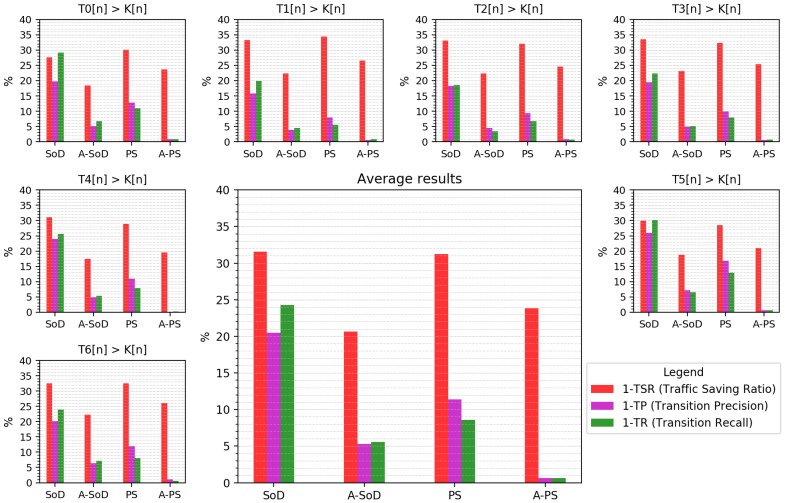
Case study III. With event-based sampling techniques SoD, PS, A-SoD, and A-PS previously detailed, the condition $T_{i}>K_{i}$ has been evaluated for each temperature signal of each station of the Arpa Piemonte repository [53]. The resulting Boolean signals are analyzed through metrics described in Section 3.2 for comparison purposes. The numeric identifier of each station in the repository is as follows: Oropa 0, Cameri 1, Alessandria 2, Vercelli 3, Pallanza 4, Montaldo Scarampi 5, and Bobes 6.

The results of the experiment can be seen in Figure 8, where there is a graph for each temperature signal of the repository, and one more with the average values of the metrics applied. Each graph shows the complementary values (for better visualization reasons) of traffic saving ratio TSR, TP, and TR (Equations (5), (9) and (10), respectively). These measurements represent the proportion of sent traffic (1−TSR), and the proportion of erroneous transitions in terms of false positives (1−TP) and false negatives (1−TR). The level accuracy metric (LP, Equation (11)) has not been represented because it has a value of 1 in all executions. This means that there are not extended periods of erroneous Boolean samples in either case. As can be observed, the graph of average results faithfully represents all specific cases. For this reason, the following observations are made on the average data of the experiment.

The average values of the experiment (central bar graph) reveal the results below. According to the proportion of traffic sent (1 − TSR), 31.5% of network packets are sent by using SoD, while this amount is reduced to 20.5% by using A-SoD, its adaptive version. The complementary values of this quantity (1−(1−TSR)) represent the TSR metric according to Equation (5), which expresses the proportion of traffic avoided. In this sense, the SoD technique achieves a Traffic Savings Ratio (TSR) of 68.5%, while A-SoD achieves 79.5%. That means an improvement of 16% in this metric. In the case of comparing PS and A-PS in the same way, the TSR values obtained are 69% and 76%, respectively, which means an increase of 10% with the adaptive version.

On the other hand, the values 1 − TP and 1 − TR in the graph represent a proportion of erroneous transitions. The first one (1 − TP) refers to false positives, i.e., transitions that occur but should not. The second (1 − TR) refers to false negatives, i.e., transitions that are expected to occur but do not. The complementary values ( 1 − (1 − TP) and 1 − (1 − TR) ) are the Transition Precision (TP, Equation (9)) and Transition Recall (TR, Equation (10)) metrics, respectively, and represent the proportion of successful transitions.

In terms of Transition Precision (TP), the comparison of SoD and A-SoD reveals an increase from 79.5% to 95%, respectively, an improvement of 19.5%. In the case of comparing PS and A-PS, TP increases from 89% to 99% respectively, an improvement of 11%. Finally, the Transition Recall (TR) obtained with SoD and A-SoD increases from 76% to 94.5% respectively, an improvement of 24%. In addition, the TR metric obtained for PS and A-PS increases from 91.5% to 99% respectively, an improvement of 8%.

It is important to emphasize the improvement achieved with the adaptability provided by the model. Especially noteworthy is the improvement in the proportions of erroneous transitions. In this aspect, using SoD, 20% of the detected transitions are erroneous, and 24% of the true transitions are not detected. With the adaptive version A-SoD, these proportions are reduced to 5%. Comparing PS and A-PS, these proportions go from 11% of erroneous detections and 8.5% of no detections, to less than 1% of both with A-PS, achieving an almost error-free system sending only 24% of the traffic.

## 6. Conclusions and Future Work

This paper proposes a data reduction model in sensor networks for a specific application: the evaluation of Boolean conditions. Within this framework, the proposal is based on two fundamental concepts, abstraction and contextualization.

Abstraction is achieved through a generic data filter definition to be implemented in the sensors, which avoids the limitation of working with real numbers. For this purpose, the proposed methodology works with data domains and reduction and equivalence functions to be defined.

Contextualization is addressed by specific metrics, which compare the original Boolean response of a system to the characteristics of the study, with the Boolean response of the system when input signals are reduced. From this comparison, relevant information about the quality of the response is extracted. From this comparison, three percentage measurements are extracted. These values describe different aspects related to the quality of the response, obtaining a more descriptive Boolean error concept.

Taking all this into account, the following hypothesis is put forward: by contextualising the use of data for Boolean calculations, more aggressive reduction techniques can be considered, achieving a more favourable cost–benefit ratio in terms of traffic savings and errors.

In order to test the proposal, two case studies and a comparative test have been carried out. For use cases, a simple quantization filter has been applied for the evaluation of a two-input condition. The input values have been taken, sample by sample, from signals of an extensive repository. Different quantizations were used to characterize the result of the system with a training data window, according to the proposed metrics. Finally, the same analysis was carried out for the rest of the data, comparing the results obtained. The result of both case studies reveals that the training window is valid for characterizing the system. This conclusion is demonstrated by the small variation in the measurements obtained with the rest of the data. These variations are in the range [0.3%, 3.1%] for TP (Table 3), [0.3%, 1.5%] for TR (Table 4), [0.3%, 1.5%] for TSR (Table 1), and [0%, 1.2%] for LP (Table 2). On the other hand, the proposed metrics provide a more accurate interpretation of the variation in system response. Specifically, the interpretation of τ reveals that erroneous samples of system response with reduced inputs tend to concentrate around transitions from the original response. In addition, the smallest τ that makes LP≃0 limits the maximum period of consecutive erroneous samples, which is an important feature of the system behavior. Case study 2 shows that the system is capable of working effectively with complex mathematical expressions, which opens the door to future developments.

On the other hand, the comparative study uses the methodology to develop adaptive techniques that maximize the savings-error ratio. In order to test the feasibility of this concept, two known event-based sampling schemes (Send–on–Delta and Predictive Sampling) and their respective adaptive versions have been implemented using the data–domain reduction for threshold–based event detection (D2R–TED) model. Here, the signals from the repository have been compared sample by sample with an increasing linear threshold that spans the entire signal range. Adaptive versions apply a simple heuristic based on a threshold distance criterion to change the reduction parameters. Despite the simplicity of the adaptability method, the results (Figure 8) show that adaptive techniques implemented using a D2R–TED model improve approximately in a range between 10% and 16% the traffic savings obtained by the non-adaptive techniques. Moreover, the Transition Precision and Recall of the A-SoD and A-PS show larger improvements from the standard methods. For instance, for Transition Precision, A-SoD improves SoD approximately in a 19.5%, and A-PS shows an improvement of 11% with PS. In Transition Recall, the improvements are 24% and 8%, for A-SoD and A-PS, respectively. Furthermore, the A-PS is able to reduce the errors almost to 0 by sending approximately 23% of network packets.

As a general conclusion, the model and the proposed metrics provide an efficient framework for the definition of data filters in sensor networks where the sink node calculates Boolean conditions. The abstraction provided by D2R–TED enables scenarios where information packages are modeled as data structures, providing the versatility inherent to computer science. The reduction and equivalence functions (Equations (3) and (4), respectively) can be redefined to establish convenient criteria in each case, opening the door to future research in areas such as vision, classification, tracking, fuzzy logic, etc. In particular, smart camera deployments can take advantage of the D2R–TED model, following a similar approach to the example shown in Figure 3.

## Figures and Tables

**Table 1:** Case study I. Traffic Saving Ratio (TSR).

	δ = 0.25	δ = 0.5	δ = 0.75	δ = 1	δ = 2.5
	0.069/0.066	**0.201/0.186**	0.307/0.291	0.393/0.373	0.677/0.666
ϵ	0.003	0.015	0.016	0.020	0.011
$\epsilon_{r}$	4.35%	7.46%	5.21%	5.09%	1.62%

**Table 2:** Case study I. Level Precision (LP).

	δ = 0.25	δ = 0.5	δ = 0.75	δ = 1	δ = 2.5	$\bar{\epsilon }$(σ)	$\bar{\epsilon_{r}}$(σ)
τ = 0	0.999/0.999	0.995/0.994	0.986/0.982	0.971/0.968	0.832/0.835	0.002 (0.001)	0.24% (0.16%)
τ = 1	1.000/1.000	0.999/0.998	0.996/0.993	0.989/0.985	0.871/0.881	0.004 (0.003)	0.39% (0.40%)
τ = 2	1.000/1.000	0.999/0.999	0.998/0.995	0.993/0.990	0.886/0.893	0.003 (0.003)	0.28% (0.29%)
τ = 3	1.000/1.000	**1.000/1.000**	0.999/0.996	0.995/0.992	0.891/0.895	0.002 (0.002)	0.21% (0.18%)
τ = 4	1.000/1.000	1.000/1.000	1.000/0.997	0.997/0.994	0.884/0.892	0.003 (0.003)	0.30% (0.33%)
τ = 5	1.000/1.000	1.000/1.000	1.000/0.998	0.998/0.994	0.871/0.884	0.004 (0.005)	0.42% (0.56%)
τ = 6	1.000/1.000	1.000/1.000	1.000/0.998	0.999/0.995	0.859/0.874	0.004 (0.006)	0.47% (0.66%)
τ = 7	1.000/1.000	1.000/1.000	1.000/0.999	1.000/0.996	0.849/0.867	0.005 (0.007)	0.52% (0.81%)
τ = 8	1.000/1.000	1.000/1.000	1.000/0.999	1.000/0.997	0.846/0.866	0.005 (0.008)	0.55% (0.91%)
τ = 9	1.000/1.000	1.000/1.000	1.000/1.000	1.000/0.998	0.848/0.868	0.004 (0.008)	0.51% (0.93%)
$\bar{\epsilon }$(σ)	0.000 (0.000)	0.000 (0.000)	0.002 (0.001)	0.003 (0.001)	0.012 (0.006)		
$\bar{\epsilon_{r}}$(σ)	0.00% (0.00%)	0.02% (0.04%)	0.22% (0.12%)	0.33% (0.06%)	1.37% (0.72%)		

**Table 3:** Case study I. Transition Precision (TP).

	δ = 0.25	δ = 0.5	δ = 0.75	δ = 1	δ = 2.5	$\bar{\epsilon }$(σ)	$\bar{\epsilon_{r}}$(σ)
τ = 0	0.910/0.906	0.743/0.748	0.555/0.600	0.474/0.491	0.189/0.200	0.016 (0.015)	3.73% (2.96%)
τ = 1	0.959/0.950	0.863/0.863	0.738/0.752	0.662/0.676	0.396/0.366	0.013 (0.010)	2.51% (2.64%)
τ = 2	0.966/0.963	0.890/0.892	0.774/0.803	0.722/0.737	0.500/0.475	0.015 (0.011)	2.27% (1.88%)
τ = 3	0.973/0.971	**0.906/0.912**	0.804/0.838	0.753/0.778	0.573/0.552	0.018 (0.012)	2.42% (1.65%)
τ = 4	0.974/0.975	0.913/0.927	0.821/0.864	0.778/0.812	0.624/0.618	0.020 (0.016)	2.44% (2.00%)
τ = 5	0.979/0.979	0.920/0.938	0.848/0.884	0.799/0.837	0.666/0.677	0.021 (0.015)	2.52% (1.75%)
τ = 6	0.980/0.981	0.930/0.947	0.861/0.896	0.820/0.857	0.707/0.725	0.022 (0.013)	2.61% (1.59%)
τ = 7	0.984/0.984	0.935/0.954	0.873/0.908	0.832/0.874	0.735/0.768	0.026 (0.015)	3.12% (1.86%)
τ = 8	0.988/0.985	0.941/0.959	0.891/0.920	0.847/0.887	0.766/0.799	0.025 (0.013)	2.90% (1.62%)
τ = 9	0.990/0.987	0.949/0.965	0.900/0.932	0.853/0.901	0.790/0.828	0.027 (0.016)	3.20% (1.96%)
$\bar{\epsilon }$(σ)	0.003 (0.002)	0.011 (0.007)	0.033 (0.008)	0.031 (0.012)	0.023 (0.010)		
$\bar{\epsilon_{r}}$(σ)	0.27% (0.01%)	1.29% (0.09%)	4.19% (0.64%)	4.23% (0.83%)	4.52% (2.61%)		

**Table 4:** Case study I. Transition Recall (TR).

	δ = 0.25	δ = 0.5	δ = 0.75	δ = 1	δ = 2.5	$\bar{\epsilon }$(σ)	$\bar{\epsilon_{r}}$(σ)
τ = 0	0.917/0.927	0.786/0.798	0.668/0.686	0.590/0.587	0.277/0.296	0.012 (0.006)	2.54% (2.28%)
τ = 1	0.967/0.973	0.913/0.921	0.888/0.860	0.825/0.807	0.580/0.541	0.020 (0.012)	2.71% (2.21%)
τ = 2	0.979/0.983	0.926/0.947	0.917/0.902	0.876/0.864	0.691/0.668	0.015 (0.007)	1.80% (0.97%)
τ = 3	0.988/0.986	**0.947/0.960**	0.937/0.924	0.904/0.897	0.755/0.738	0.010 (0.005)	1.20% (0.69%)
τ = 4	0.989/0.989	0.954/0.968	0.947/0.940	0.916/0.919	0.790/0.784	0.006 (0.005)	0.66% (0.49%)
τ = 5	0.991/0.990	0.964/0.973	0.954/0.949	0.937/0.931	0.836/0.817	0.008 (0.006)	0.89% (0.74%)
τ = 6	0.991/0.991	0.974/0.977	0.960/0.956	0.950/0.943	0.852/0.844	0.004 (0.003)	0.48% (0.33%)
τ = 7	0.992/0.993	0.974/0.981	0.963/0.964	0.955/0.952	0.869/0.863	0.004 (0.002)	0.39% (0.27%)
τ = 8	0.995/0.994	0.978/0.985	0.968/0.970	0.963/0.960	0.884/0.879	0.004 (0.002)	0.38% (0.23%)
τ = 9	0.996/0.995	0.982/0.988	0.975/0.974	0.967/0.967	0.898/0.892	0.003 (0.003)	0.30% (0.28%)
$\bar{\epsilon }$(σ)	0.003 (0.003)	0.010 (0.005)	0.009 (0.008)	0.006 (0.005)	0.015 (0.010)		
$\bar{\epsilon_{r}}$(σ)	0.27% (0.01%)	1.07% (0.07%)	1.04% (0.13%)	0.71% (0.12%)	2.24% (1.07%)		

**Table 5:** Case study II. Traffic Saving Ratio (TSR).

	δ = 0.25	δ = 0.5	δ = 0.75	δ = 1
	0.062/0.069	0.194/0.211	0.332/0.342	0.430/0.440
ϵ	0.007	0.017	0.010	0.010
$\epsilon_{r}$	11.29%	8.76%	3.01%	2.33%
